# Smac mimetic LCL161 overcomes protective ER stress induced by obatoclax, synergistically causing cell death in multiple myeloma

**DOI:** 10.18632/oncotarget.11028

**Published:** 2016-08-02

**Authors:** Vijay Ramakrishnan, Marcus Gomez, Vivek Prasad, Teresa Kimlinger, Utkarsh Painuly, Bedabrata Mukhopadhyay, Jessica Haug, Lintao Bi, S. Vincent Rajkumar, Shaji Kumar

**Affiliations:** ^1^ Division of Hematology, Mayo Clinic, Rochester, MN, USA; ^2^ 4th Department of Internal Medicine-Hematology, University Hospital Hradec Kralove and Charles University in Prague, Faculty of Medicine in Hradec Kralove, Czech Republic; ^3^ The Department of Hematology and Oncology, China-Japan Union Hospital, Jilin University, Changchun, Jilin, China

**Keywords:** IAP, Bcl-2, myeloma, apoptosis, GRP78

## Abstract

Bcl2 and IAP families are anti-apoptotic proteins deregulated in multiple myeloma (MM) cells. Pharmacological inhibition of each of these families has shown significant activity only in subgroups of MM patients. Here, we have examined a broad-spectrum Bcl2 family inhibitor Obatoclax (OBX) in combination with a Smac mimetic LCL161 in MM cell lines and patient cells. LCL161/OBX combination induced synergistic cytotoxicity and anti-proliferative effects on a broad range of human MM cell lines. The cytotoxicity was mediated through inhibition of the IAPs, activation of caspases and up regulation of the pro-apoptotic proteins Bid, Bim, Puma and Noxa by the drug combination. In addition, we observed that OBX caused ER stress and activated the Unfolded Protein Response (UPR) leading to drug resistance. LCL161, however inhibited spliced Xbp-1, a pro-survival factor. In addition, we observed that OBX increased GRP78 localization to the cell surface, which then induced PI3K dependent Akt activation and resistance to cell death. LCL161 was able to block OBX induced Akt activation contributing to synergistic cell death. Our results support clinical evaluation of this combination strategy in relapsed refractory MM patients.

## INTRODUCTION

Novel therapies have greatly improved the prognosis of multiple myeloma (MM) patients [1]. Yet, patients continue to relapse and become refractory to existing therapies. Hence, there is an urgent need for newer therapies based on a better understanding of the disease biology. Apoptotic pathways are deregulated in hematological malignancies including MM and contributes towards resistance observed, to existing therapies [2], Inhibiting these anti-apoptotic pathways therefore holds considerable promise to overcoming resistance in MM.

The Bcl-2 family of proteins includes multiple pro and anti-apoptotic members that are characterized by the presence of one or more Bcl-2 homology (BH) domains [3, 4]. The ratio of the pro and anti-apoptotic proteins and the complex interactions between them determines whether an individual cell survives or undergoes apoptosis [3, 4]. The anti-apoptotic Bcl-2 family members include Bcl-2, Bcl-Xl, Bcl-w and Mcl-1, all of which contain multi BH domains (BH1-4). The pro-apoptotic Bcl-2 family members can be further grouped into sensitizer, activator, and effector proteins. The sensitizer and activator proteins are both single BH domain (BH3) -containing proteins that modulate the activity of the effector proteins through different mechanisms. The sensitizer proteins include Bad, Bik, Bmf, Puma and Noxa and the activator proteins are Bid and Bim. The effector proteins Bak and Bax, which are both multi BH domain-containing proteins, specifically cause mitochondrial membrane permeabilization thereby releasing pro-apoptotic proteins into the cytosol [3, 4].

In MM, the anti-apoptotic Bcl-2 family members have been shown to be up regulated and important for MM cell survival and resistance to existing therapies [5–8]. Molecules targeting the interaction between pro and anti-apoptotic Bcl-2 proteins have shown significant pre-clinical activity [9–12]. Recently, it has been shown that molecules inhibiting Bcl-2, but not Mcl-1, show promising anti-MM activity only in MM cell lines and patients with t(11;14) translocation, the same group that expresses high levels of both Bcl-2 and Bcl-Xl [13, 14]. Obatoclax (OBX), a molecule inhibiting Mcl-1, Bcl-2 and Bcl-Xl has shown potent activity against cytogenetically distinct MM cell lines and patient cells alone or in combination with other agents [9, 15]. However, limited activity was seen after OBX treatment in a clinical trial involving MM patients [16].

Inhibitor of apoptosis proteins (IAPs) represent another important family of anti-apoptotic proteins, comprising of eight family members, and is potentially a therapeutic target in MM [17]. IAPs inhibit apoptosis by blocking activation of caspases [18, 19]. The three most well studied IAP proteins are cellular IAP 1 (cIAP1), cellular IAP 2 (cIAP2) and X-linked IAP (XIAP). Of these three, XIAP binds directly to caspases 9 and 3 and blocks their activation [20]. cIAP-1 and cIAP-2, on the other hand, indirectly regulate the extrinsic apoptotic pathway through ubiquitination of receptor-interacting protein (RIP) [21, 22]. During conditions that signal cells to commit suicide, Second Mitochondria-derived Activator of caspases (Smac) is released from the mitochondria. Once in the cytosol, Smac binds to IAPs at Smac binding sites thereby preventing their activity [23, 24]. However, many cancers including MM express aberrantly high levels of IAPs, thus successfully blocking apoptosis in tumors [17, 25–27]. We recently showed that IAP inhibition by using the Smac mimetic LCL161 induced potent apoptosis in a subset of MM cell lines and patient cells [17]. In that study, we observed that the Bcl-2 family of anti-apoptotic proteins was not down regulated post LCL161 treatment [17]. It has also been shown that Bcl-2 expression confers resistance to LCL161 treatment in hepatocellular carcinoma [28]. We therefore hypothesized that combining a Smac mimetic such as LCL161 with a pan Bcl-2 family inhibitor such as OBX would overcome the apoptotic resistance of MM cells and trigger them to undergo programmed cell suicide.

In this study, we observed that LCL161 in combination with OBX led to increased apoptotic activity, inducing cell death synergistically and greatly reducing proliferation. Upon further examination of the mechanism of action of the drug combination, we observed activation of the endoplasmic reticulum (ER) stress response by OBX. OBX caused ER stress led to cell survival signals mediated in part by GRP78 induced Akt activation. LCL161 modulated the ER stress response by reducing levels of spliced Xbp1, a survival factor implicated in MM. LCL161 also reduced OBX induced pAkt thereby contributing to enhanced apoptosis.

## RESULTS

### LCL161 and OBX synergistically promote cytotoxicity, inhibit proliferation, and overcome protective shield of the microenvironment in MM

**Figure 1:**
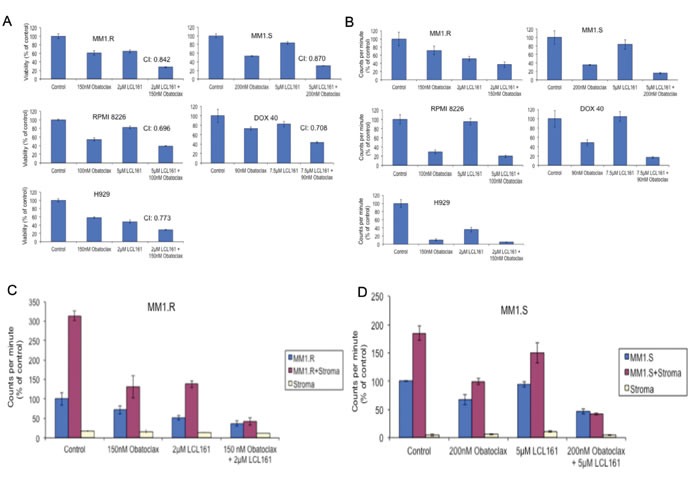
When MM cell lines were treated with LCL161, OBX or the combination for A. 72hrs and B. 48hrs, we observed **A.** synergistic cytotoxicity when cells were treated with LCL161/OBX combination and **B.** greater inhibition of proliferation when cells were treated with LCL161/OBX combination. **C.** MM1.R and MM1.S cells were cultured with or without bone marrow stromal cells (BMSCs) derived from a MM patient. Cells were treated with indicated concentrations of LCL161, OBX or the combination for 48hrs. We observed that only LCL161/OBX combination, but not the individual drugs, was able to overcome the tumor protective effects of BMSCs and inhibit proliferation to the extent observed when the MM cells were cultured alone in the absence of BMSCs.

First, we tested if LCL161/OBX combination induced synergistic cytotoxicity in MM cells. For this, MM cell lines were incubated with varying concentrations of both single agent and combination of drugs for 72 hours. Out of 9 lines tested, 6 showed a synergistic relationship (CI < 1) between the LCL161 and OBX, reaching IC-50 levels at notably lower doses of the individual drugs compared with that used in combination. The concentrations and the cytotoxicity at the combination with maximum synergy (lowest CI value) are shown (Figure 1A). Proliferation assays after incubation with both agents yielded similar results, with the anti-proliferative effect of the combination treatment being notably higher in comparison to single agent effects (Figure 1B).

Next, we examined if the synergistic anti-tumor effects elicited by LCL161/OBX was also observed when MM cells were co-cultured with components of the microenvironment. For this, we co-cultured MM cell lines with bone marrow stromal cells (BMSCs) from MM patients and then treated with LCL161, OBX or the combination. We observed that the combination treatment was able to overcome the protective effects of the tumor-promoting microenvironment more effectively than either of the agents individually (Figures 1C).

### LCL161 and OBX promote apoptotic cell death

**Figure 2:**
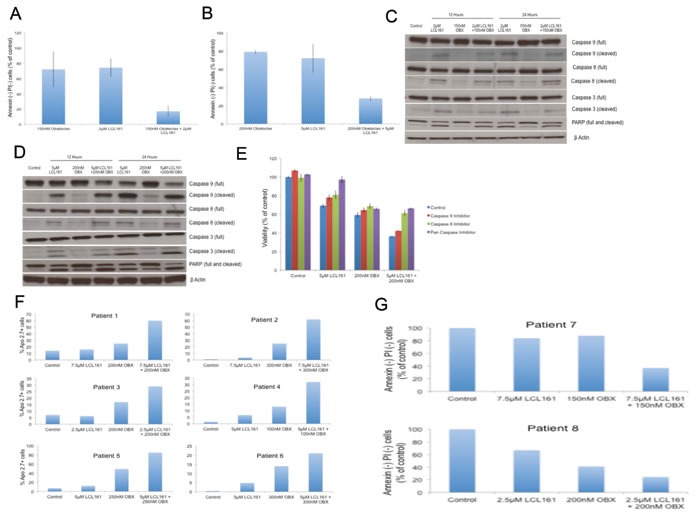
**A.** MM1.R was incubated with 2μM of LCL161, 150nM of OBX or the combination for 72hrs. **B.** MM1.S was incubated with 5μM of LCL161, 200nM of OBX or the combination for 72hrs. In both **A.** and **B.** apoptosis induction was measured using Annexin/PI staining and in both cases the drug combination induced more apoptosis than either of the individual drugs. **C.** MM1.R and **D.** MM1.S cells were incubated with indicated concentrations of LCL161, OBX or the combination for 12 or 24 hours and caspases 9, 8 and 3 and PARP levels were examined by western blotting. β Actin was used as a loading control. All experiments were performed thrice and the results presented are from a representative experiment. **E.** We incubated MM1.S cells with indicated doses of LCL161, OBX or the combination for 72hrs alone or in the presence of 10μM of either caspase 9 inhibitor (Ac-LEHD-CMK), caspase 8 inhibitor (Z-IETD-FMK) or pan-caspase inhibitor (Q-VD-OPH) and measured the cytotoxicity induced by MTT assays. **F.** Primary cells from 6 MM patients were incubated with indicated doses of LCL161, OBX or the combination for 72 hrs. Apo 2.7 staining was done to examine apoptosis induction post drug treatment. **G.** Primary cells from 2 MM patients were incubated with indicated doses of LCL161, OBX or the combination for 72hrs. Annexin/PI staining was done to examine apoptosis induction post drug treatment.

We then examined if LCL161/OBX caused cell death through the induction of apoptosis. For this and for our further studies, we used two MM cell lines, one sensitive (MM1.R) and the other relatively resistant to LCL161 (MM1.S) [17]. These are isogenic cell lines derived from a parental cell line (MM1), with one being sensitive (MM1.S) and the other being resistant to dexamethasone (MM1.R) [29]. We used these genetically similar cell lines in our studies with the hope of identifying factors that could predict for sensitivity to LCL161 in addition to examining the mechanism of action of LCL161/OBX combination. MM1.R and MM1.S were incubated with LCL161, OBX or the combination and apoptosis was examined using the Annexin-PI assay. Our results indicated that while the drugs induced apoptosis as single agents, the combination clearly induced more pronounced apoptosis in both the cell lines (Figures 2A and 2B). Furthermore, western blots revealed increased activation of caspases 8, 9, and 3 and PARP cleavage post LCL161 or the combination treatment indicating activation of both extrinsic and intrinsic apoptotic pathways by LCL161 or the combination (Figures 2C and 2D). From Figures 2C and 2D, it is clear that OBX at the doses used is unable to induce activation of caspases. To further confirm the role of caspases in the apoptosis induced by the drugs, we incubated MM1.S with LCL161, OBX or the combination along with caspase inhibitors. We observed that the ability of LCL161 to induce cell death was completely blocked by pan-caspase inhibitor (Figure 2E). The activity of OBX was not significantly influenced by any of the caspase inhibitors. This suggests that caspase activation is not essential for OBX induced cell death at the doses used in this study. Synergy between the drugs was not observed when cells were treated with caspase 8 or pan caspase inhibitors. Thus, the ability of LCL161 to induce caspase 8 dependent extrinsic apoptosis appears to be essential for the observed synergy between the drugs.

Trudel et al [9] showed that OBX induces caspase activation in MM cells albeit at doses much higher than the ones used here in our study. Thus, caspase activation appears to be triggered by OBX at higher doses and might be required to induce more pronounced and complete cell death. However, here we were able to induce pronounced cell death by using OBX at lower doses in combination with LCL161. LCL161 through direct IAP inhibition or through other mechanisms is able to overcome such resistance mechanisms to OBX and induce synergistic cell death.

Next, we examined the effects of LCL161/OBX combination on primary plasma cells from 8 MM patients and observed that the drug combination induced more potent apoptosis than either of the single agents (Figures 2F and 2G).

### LCL161 and OBX effectively inhibit IAPs and up-regulate pro-apoptotic Bcl-2 family proteins

**Figure 3:**
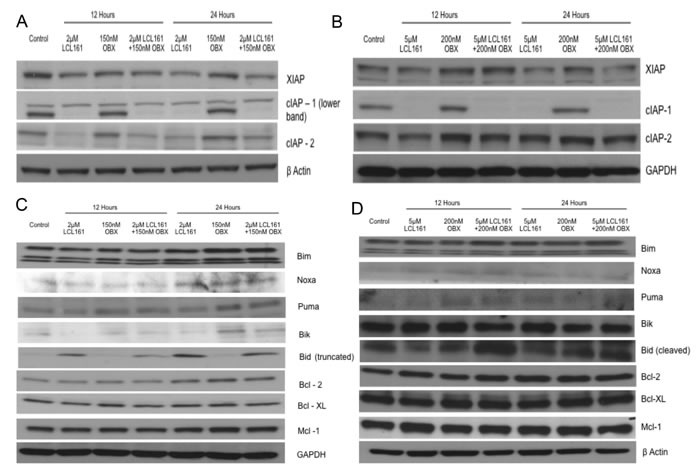
**A.** MM1.R and **B.** MM1.S cells were incubated with indicated concentrations of LCL161, OBX or the combination for 12 or 24hours and XIAP, cIAP1 and cIAP2 levels were examined using western blots. β Actin or GAPDH was used as a loading control respectively. **C.** MM1.R and **D.** MM1.S cells were incubated with indicated concentrations of LCL161, OBX or the combination for 12 or 24 hours and expression levels of the Bcl2 family of pro and anti-apoptotic proteins were observed. GAPDH or β Actin was used as a loading control respectively. All experiments were done three times. Results are from one representative experiment.

From the results depicted in Figures 2A-2D, it became clear that the LCL161/OBX combination induces apoptosis. We wanted to understand the mechanism through which apoptosis was induced by the drug combination. LCL161 induced down regulation of cIAP1 and XIAP in both MM1.R and MM1.S cells. LCL161 was unable to cause a significant down regulation of cIAP2 in MM1.S cells as reported earlier [17] (Figures 3A and 3B). Furthermore, pro-apoptotic Bcl-2 family proteins Bim, and Puma were up regulated by OBX, while Bid was up regulated by LCL161 (Figures 3C and 3D). In addition, we also saw Noxa up regulation by both LCL161 and OBX in both MM1.R and MM1.S cells and Bik up regulation by OBX in MM1.R cells (Figures 3C and 3D). Importantly, all these effects were retained when the cells were treated with the combination.

### OBX induces protective autophagy

From the results presented in Figures 2C, 2D and 2E, we hypothesized that the lack of caspase activation and more pronounced apoptosis by OBX was due to the fact that the cells induced resistance mechanism(s) to the drug treatment. More importantly, this also suggested that LCL161 was able to overcome such resistance mechanisms and induce synergistic apoptosis.

**Figure 4:**
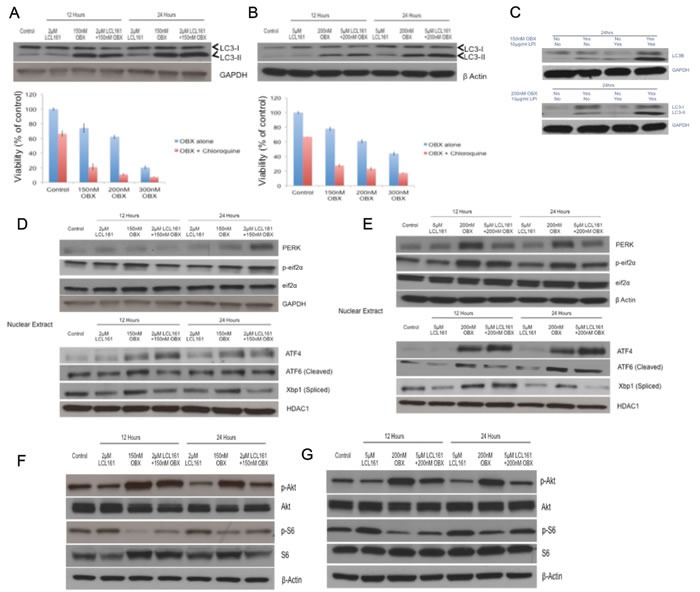
Upper panel **A.** and **B.** MM1.R and MM1.S cells were incubated with indicated concentrations of LCL161, OBX or the combination for 12 or 24hours and LC3-I and II levels were examined. Lower panel **A.** and **B.** MM1.R and MM1.S cells were incubated with indicated concentrations of OBX for 72hrs alone or in the presence of 20μM chloroquine and cytotoxicity was measured by MTT assays. **C.** When MM1.R or MM1.S cells were treated with indicated concentrations of OBX alone, 10μg/ml of lysosomal protease inhibitors (LPI) alone or the combination for 24hrs, we observed increased accumulation of LC3-II when cells were treated with OBX in combination with LPI. Experiments were performed thrice and results from a representative experiment are shown. Upper panel **D.** and **E.** MM1.R and MM1.S cells were incubated with indicated concentrations of LCL161, OBX or the combination for 12 or 24hours and PERK and p-eif2α levels were examined by western blots. GAPDH or β Actin was used as a loading control. Lower panel **D.** and **E.** MM1.R and MM1.S cells were incubated with indicated concentrations of LCL161, OBX or the combination for 12 or 24hours. Following incubation, nuclear extracts were made and expression levels of ATF4, ATF6 (cleaved) and Xbp1 (spliced) were examined by western blots. HDAC1 was used as a loading control. Experiments were performed three times and results from a representative experiment are shown. **F.** MM1.R and **G.** MM1.S cells were incubated with indicated concentrations of LCL161, OBX or the combination for 12 or 24hours and pAkt and pS6 levels were examined using western blots. β Actin was used as a loading control. Experiments were performed three times and results from a representative experiment are shown.

Given that OBX could induce autophagy [30, 31], we wanted to examine if OBX induced autophagy as a mechanism to overcome cell death signals. For this, we examined levels of microtubule-associated protein 1A/1B-light chain 3 (LC3) levels. The conversion of LC3-I to the lipidated LC3-II form is a marker of autophagy [32]. Examining LC3 levels clearly showed accumulation of LC3-II after OBX treatment indicating induction of autophagy (Figures 4A and 4B). To identify if the autophagy induced was cytoprotective, we treated MM1.R and MM1.S cells with OBX, autophagy inhibitor chloroquine or OBX in combination with chloroquine. Chloroquine treatment sensitized both cell lines to OBX activity, suggesting that OBX induced protective autophagy (Figures 4A and 4B). Using chloroquine in combination with LCL161 did not show any significant increase in cell death (data not shown). To confirm if LC3-II accumulation was due to autophagy induction and not due to blockage of it's autophagic degradation [33], we treated cells with OBX in the presence or absence of lysosomal protease inhibitors (LPI). Our results showed increased LC3-II accumulation in the presence of LPI confirming induction of autophagy (Figure 4C).

### LCL161 and OBX modulate different arms of the Endoplasmic Reticulum (ER) stress induced unfolded protein response (UPR) pathway

Given that autophagy could be induced through the unfolded protein response (UPR) pathway, we wanted to understand if OBX modulated this pathway. In a recent study, it was shown that OBX induced the UPR pathway, which contributed to cell survival [34]. We therefore examined expression levels of proteins involved in the UPR pathway. We observed clear modulation of ER stress mediated UPR pathway by OBX and surprisingly by LCL161 as well. However, LCL161 and OBX targeted different branches of the UPR pathway. OBX activated the ATF6 and PERK/peif2α/ATF4 branches of the UPR (Figures 4D and 4E), both of which have been implicated in cell survival during ER stress [35, 36]. ATF4 under irrecoverable ER stress can lead to increase in transcription of CHOP and cause apoptosis. We therefore examined levels of CHOP and observed no induction of CHOP post treatment with either of the drugs or the combination (data not shown). LCL161, however inhibited the IRE1 branch of the UPR by down regulating Xbp-1 splicing [37], which is a pro survival activity of IREI [37], suggesting that LCL161 targeted ER stress to induce apoptosis (Figures 4D and 4E).

### OBX causes GRP78 migration to the plasma membrane and activates Akt

Next, we wanted to examine how LCL161/OBX modulated other signaling pathways important for MM cell survival and observed that pAkt (Ser 473) was up regulated by OBX (Figures 4F and 4G). To our knowledge, this is the first report showing pAkt up regulation post OBX treatment. Surprisingly, we found that OBX down regulated activated levels of S6, a protein downstream of mTOR (Figures 4F and 4G). It was interesting to note that LCL161 when used in combination with OBX was able to prevent both the increase in pAkt and the decrease in pS6 induced by OBX (Figures 4F and 4G).

**Figure 5:**
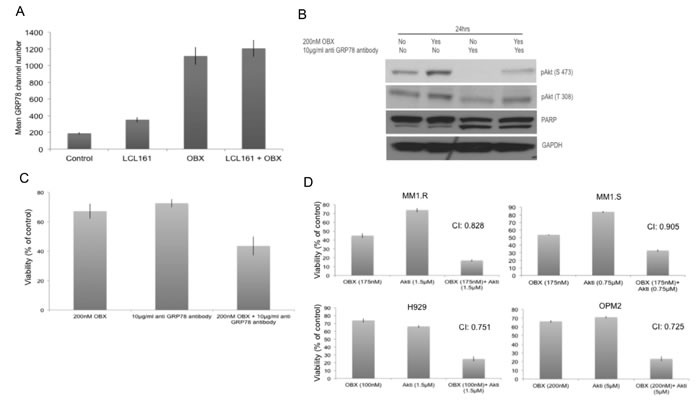
**A.** MM1.R was left untreated or treated with 150nM OBX, 2μM LCL161 or the combination for 12hrs and cell surface expression of GRP78 was examined using flow cytometry. Y-axis represents mean GRP78 expression channel number. OBX has high auto fluorescence and hence results are presented as mean GRP78 expression channel number, which is the difference between mean channel number with GRP78 antibody and the mean channel number without GRP78 antibody for each treatment condition. Experiments were performed two times and error bars represent one standard deviation. **B.** MM1.R cells were treated with indicated concentrations of OBX, anti-GRP78 antibody (polyclonal antibody) or the combination and pAkt (S473), pAkt (T308) and PARP levels were examined by western blotting. GAPDH was used as a loading control. Experiments were performed two times and results from a representative experiment are shown. **C.** MM1.R cells were treated with indicated concentrations of OBX, anti GRP78 antibody (Clone C38) or the combination for 72hrs following which viability was measured by a MTT assay. OBX+GRP78 induced significantly more cell death than either of the agents alone (*p* < 0.05). The results presented are the mean of 5 independent experiments. **D.** MM1.R, MM1.S, H929 and OPM2 cells were treated with various concentrations of OBX for 72hrs, various concentrations of Akti for 48hrs or the drugs in combination. MTT assays were performed. Synergy was seen across multiple concentrations. The concentrations at which maximum synergy was observed is shown in the figure. Experiments were performed three times.

We then wanted to understand how OBX induced pAkt. GRP78, an ER chaperone protein is expressed on the surface of malignant cells including MM cells and activates signal transduction pathways thereby promoting their survival [38, 39]. In another study, it was shown that cancer cells resistant to therapy promote GRP78 migration to the cell membrane, which was further enhanced by ER stress [40]. Given that OBX induced ER stress and Akt activation, we hypothesized that OBX promoted GRP78 migration to the cell membrane, which subsequently caused Akt activation. To test this, we first examined cell surface expression of GRP78 after treating MM cells with OBX, LCL161 or the combination. We observed clear increase in cell surface expression of GRP78 after treating cells with OBX or OBX+LCL161 (Figure 5A). To understand if increased cell surface localization of GRP78 contributed to the activation of Akt by OBX, we treated MM cells with an antibody to GRP78 (clone C38) that has been shown to block the Akt activation by GRP78 [41]. Our results clearly show down regulation of both pAkt (T308) and pAkt (S473) by the anti-GRP78 antibody. More importantly, our results also showed the ability of the antibody to reduce OBX induced pAkt levels when used in combination with OBX (Figure 5B). Next, we wanted to examine if the pAkt up regulation contributed to resistance to OBX treatment. For this, we treated cells with OBX, anti-GRP78 (clone C38) or the combination and observed significantly more cell death when the cells were treated with the antibody drug combination (Figure 5C). Using the antibody in combination with LCL161 did not show enhanced cell death (data not shown) further suggesting that GRP78 mediated pAkt up regulation was a specific resistance factor to OBX. Furthermore, synergistic cell death was also observed when OBX was used in combination with an Akt inhibitor (Figure 5D) or a PI3K inhibitor (data not shown). Thus, our results suggest that OBX caused GRP78 localization to the cell surface, which then led to activation of pAkt in a PI3K dependent mechanism. We then checked to see if GRP78 associated directly with PI3K and observed no detectable association between the proteins (data not shown).

## DISCUSSION

During early stages, MM plasma cells are relatively more proliferative and dependent on the microenvironment both of which decrease with disease progression. Plasma cells in advanced myeloma patients are typically geared towards long-term survival and low apoptotic rates. Alterations in the anti/pro-apoptotic protein ratio are an important contributing factor for the low apoptotic rates as well as for resistance observed to existing therapies. Inhibiting these anti apoptotic pathways is therefore of clinical relevance in MM. Two important apoptotic pathways that are de regulated in MM are the ones mediated by the Bcl-2 and IAP families. Inhibiting either one of the pathways alone appears to show significant response only in a limited set of MM cell lines and patient cells [13, 14, 17]. Moreover, inhibiting the Bcl-2 family using OBX showed significant neurotoxicity in a clinical trial in MM [16]. Using OBX in combination with other agents therefore promises to be able to reduce the toxicities while still significantly inducing apoptosis in MM cells. Our earlier studies using LCL161 identified up regulated levels of pStat3 and NF-κB post drug treatment, both of which can modulate expression levels of Bcl-2 family of proteins [17]. Here, we simultaneously suppressed both these protein families and observed potent synergy when the drugs were used in combination.

In addition to OBX, which is a pan-Bcl-2 family inhibitor, ABT-737 and ABT-199 are two other drugs inhibiting the Bcl-2 family that have been investigated in MM. ABT-737 is a Bcl-2, Bcl-Xl and Bcl-w inhibitor while ABT-199 is a Bcl-2 inhibitor. We used LCL161 in combination with either ABT-737 or ABT-199 and found that LCL161 did not synergize with ABT-737 or ABT-199 (data not shown) indicating that Mcl-1 inhibition could be important for the observed synergy between LCL161 and OBX. We also observed induction of Mcl-1 binding partners Bim, Noxa and Puma [42] after OBX treatment and the combination, further suggesting Mcl-1 inhibition It has been shown in a prior study that OBX was able to inhibit Mcl-1/Bak interaction but not Bcl-2/Bak interaction in MM cells (Trudel et al) further suggesting that OBX induced pro-apoptotic Bim, Noxa and Puma up regulation is mediated through Mcl-1 inhibition. However, OBX did not cause activation of caspases, nor did it induce caspase dependent cell death suggesting that mechanisms independent of Mcl-1 inhibition could be involved in cell death induced by the drug. OBX has been shown to induce autophagy that can be either cytoprotective or cytotoxic to cells [30, 31]. We observed that OBX induced protective autophagy in MM cells.

**Figure 6:**
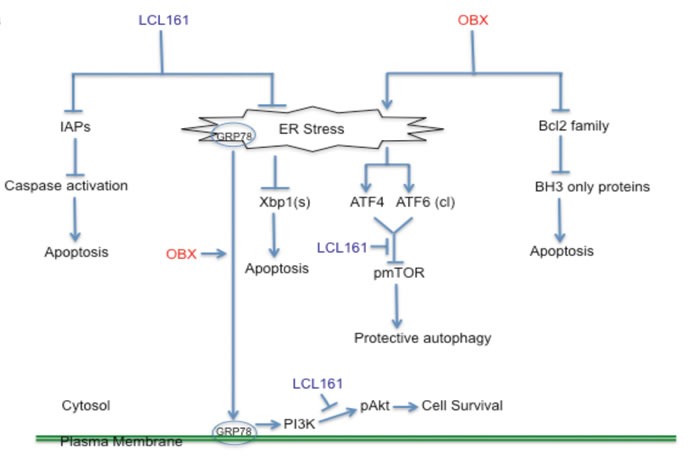
A model depicting the mechanism of action of LCL161/OBX in MM cells LCL161 binds and inhibits the functions of IAPs causing activation of caspases and apoptosis induction. OBX modulates the binding of the pro-apoptotic Bcl2 family proteins with the anti- apoptotic Bcl2 family proteins and causes the activation of BH3 only proteins, which eventually leads to apoptosis induction. In addition, both OBX and LCL161 modulate ER stress and the subsequent activation of specific branches of the UPR pathway. OBX activates the PERK/peif2α/ATF4 and ATF6 branches of the UPR pathway. One or both of these possibly contribute to pmTOR inhibition, which leads to protective autophagy. OBX also causes cell surface localization of GRP78, which then activates Akt through a PI3K dependent mechanism. LCL161, on the other hand, causes reduction in Xbp1 splicing. In addition, LCL161 blocks OBX induced pAkt, which then sensitizes cells to OBX treatment. We also observed that pS6 (a protein downstream of mTOR) down regulation by OBX was largely prevented by LCL161. Thus, LCL161/OBX combination induces synergistic apoptosis in MM cells through numerous mechanisms.

Other studies have shown the UPR pathway activation as important factors for MM cell survival, and agents perturbing this pathway induce cell death in myeloma [43, 44]. Moreover, it has been shown that OBX can induce ER stress [34, 45]. Our studies showed the activation of ER stress induced UPR pathways by both OBX and LCL161 in MM cells. OBX induced recoverable ER stress that led to activation of survival mechanisms However, LCL161 was able to counteract this resistance mechanism by inhibiting spliced Xbp1 levels and pAkt down regulation. When we examined levels of other important signaling pathways implicated in MM, we observed that OBX caused an up regulation of pAkt (Ser 473). A prior study showed that Akt is activated *via* ER stress induced after treatment with thapsigargin or tunicamycin [46]. In the same study, the authors showed that the activated Akt led to up regulation of IAPs, which caused resistance to ER stress induced apoptosis and ablation of the IAPs led to increased sensitivity to these agents [46]. In our study, we show that OBX promoted cell surface localization of GRP78, which then led to activation of Akt through PI3K dependent pathways. To confirm that GRP78 induced pAkt up regulation contributed to resistance to OBX, we used an antibody to GRP78 or an Akt1/2 inhibitor (Akti) in combination with OBX. Both combinations promoted cell killing by OBX suggesting that this was indeed a resistance mechanism to OBX. Interestingly, LCL161 was able to reduce both baseline as well as OBX induced pAkt though it did not reduce cell surface localization of GRP78. In addition, we also observed down regulation of pS6 suggesting mTORC1 inhibition after OBX treatment. We speculate that mTORC1 inhibition occurs through the ATF4 or ATF6 arms of the UPR induced by OBX. Interestingly, LCL161 was able to largely prevent OBX mediated pS6 down regulation. Taken together, LCL161/OBX induced synergistic cell death is mediated through several mechanisms as shown in Figure 6. Our study is also on similar lines as recent studies that have shown GRP78 as a therapeutic target in MM [39, 47, 48] and presents clear evidence that GRP78 activates PI3K/Akt signaling in MM, a pathway important for MM cell survival and resistance to existing therapies. Targeting GRP78 in combination with existing therapies might therefore show promising activity in MM cells.

## MATERIALS AND METHODS

### MM cell lines and patient cells

Cell lines MM1.S (Dexamethasone sensitive), MM1.R (Dexamethasone resistant) and RPMI 8226 were kindly provided by Dr. Jonathan Keats (TGen, Phoenix, AZ, USA), DOX 40 (Doxorubicin resistant) was kindly provided by Dr. William Dalton (Moffitt Cancer Center, Tampa, FL, USA), and NC-H929 was purchased from ATCC (Manassas, VA, USA). Cells were cultured in RPMI 1640 media (Mediatech Inc., Manassas, VA, USA) containing 10% fetal bovine serum (Mediatech Inc.), 100U/mL penicillin, 100uG/ml streptomycin, and 2mM glutamine (Invitrogen, Grand Island, NY, USA). Freshly obtained bone marrow aspirates from MM patients were obtained after informed consent and processed to collect either myeloma cells or bone marrow stromal cells (BMSCs) as described earlier [49–51]. Patient cells were cultured as MM cell lines except that 20% fetal bovine serum was used instead of 10% fetal bovine serum.

### Drugs and cytokines

LCL161 was synthesized and provided by Novartis (Basel, Switzerland) under a Material Transfer Agreement (MTA). Obatoclax was purchased from Selleckchem (Houston, TX, USA). Pancaspase inhibitor (Q-VD-OPH), caspase 9 inhibitor (Ac-LEHD-CMK) and caspase 8 inhibitor (Z-IETD-FMK) were purchased from EMD Millipore (Billerica, MA, USA). Akti and chloroquine were purchased from Sigma-Aldrich (St. Louis, MO, USA).

### Cytotoxicity and proliferation assays

MM cell lines were cultured in 96-well culture plates with either the single agent drugs or combination. To measure cytotoxicity, a 3- [4,5-dimethylthiazol-2-yl]-2,5 diphenyl tetrazolium bromide (MTT) colorimetric assay was employed [49–51]. For MTT experiments with GRP78, the antibody used was purchased from EMD Millipore (Clone C38 Cat # MABS443). To measure proliferation, tritiated thymidine (3H-TdR) uptake was measured [49–51]. For experiments involving co-culture with BMSC, cell lines were cultured with or without prior co-culture agents for 48 hours, and the aforementioned thymidine uptake assay was employed.

### Apoptosis assays

Apoptotic activity in MM cell lines was measured using Annexin-V/PI assay. Cells were cultured with either single agent drugs or combination treatment for 48 or 72hrs. Cells were harvested and washed once in annexin-binding-buffer (ABB) comprised of 10 mM HEPES pH 7.4, 140 mM NaCl, 2.5 mM CaCl_2_. Cells were incubated with 3μL of Annexin-FITC for 15 minutes in the dark at room temperature. The volume was brought to 2mL of ABB, and cell suspensions were spun at 400g for 5mins. Pellets were resuspended in 0.5mL of ABB with 5μL of 1μg/μL propidium iodide (PI) (Sigma-Aldrich). Samples were run on a Canto 2 flow cytometer (BD Biosciences, San Jose, CA, USA). Viable cells were characterized by Annexin/PI negative staining. In addition, apoptotic activity in MM patient derived primary plasma cells was measured using Apo 2.7 assay as described earlier [49, 52].

### Nuclear Extracts

Nuclear extracts were prepared using the NE-PER Nuclear extraction kit (Thermo Fisher Scientific, Rockford, IL, USA) following manufacturer's protocol. Following extraction, BCA assay was used to calculate protein concentration (Thermo Fischer Scientific) and samples were then used for western blots as described below.

### Western blots

Western blots were performed as detailed elsewhere [49–52]. All antibodies used except the ones specified below were purchased from Cell Signaling Technology (Danvers, MA, USA). cIAP1 antibody was obtained from Epitomics (Burlingame, CA, USA). Puma, ATF6 and XBP1 antibodies were purchased from Santa Cruz Biotechnology (Dallas, TX, USA) and Noxa antibody was purchased from EMD Millipore. Stripping buffer was purchased from EMD Millipore. Antigen-antibody complexes were detected using enhanced chemiluminescence (GE Healthcare, Piscataway, NJ, USA).

### GRP78 surface expression

After treatment with drugs, cells were harvested, pelleted and washed with PBS. Subsequently, the pellet was split into two tubes. To tube 2, 10μl of a 1:50 dilution (PBS/3%BSA) of GRP78 antibody (Cat # pa511418 from Thermo Fisher Scientific, Waltham, MA) was added and tube 1 was the no antibody control. Tubes were incubated for 15-30 minutes at room temperature. 2ml of PBS was added to each tube and they were spun for 5 minutes. Supernatants were decanted and 100μl of a 1:250 dilution (PBS/3% BSA) of goat anti mouse IgG (fab specific- Sigma F-4018) was added to each tube and incubated for 15-30 minutes at room temperature, in the dark followed by a wash with PBS. Supernatants were decanted and cells were resuspended in 0.5ml of 1% paraformaldehyde. Tubes were kept in the dark until run on the BD CantoII flow cytometer.

### Isobologram analysis

Synergy was quantified in the MTT cytotoxicity analyses utilizing the program Calcusyn. By the Chou-Talalay method, combination index values (CI values) were calculated by the equation CI = (D1)/(Dx1)+(D2)(Dx2) +(D1)(D2)/(Dx1)(Dx2), where D1 and D2 are the doses of drugs 1 and 2 required to achieve x effect in combination, and Dx1 and Dx2 are the doses of drugs 1 and 2 needed to achieve the same x effect as single agents [53].
